# Water Quality Assessment in the Harbin Reach of the Songhuajiang River (China) Based on a Fuzzy Rough Set and an Attribute Recognition Theoretical Model 

**DOI:** 10.3390/ijerph110403507

**Published:** 2014-03-26

**Authors:** Yan An, Zhihong Zou, Ranran Li

**Affiliations:** School of Economics and Management, Beihang University, Beijing 100191, China; E-Mails: anyanmog@163.com (Y.A.); liranran1101@163.com (R.L.)

**Keywords:** fuzzy rough set, attribute recognition theoretical model, attribute reduction, water quality assessment

## Abstract

A large number of parameters are acquired during practical water quality monitoring. If all the parameters are used in water quality assessment, the computational complexity will definitely increase. In order to reduce the input space dimensions, a fuzzy rough set was introduced to perform attribute reduction. Then, an attribute recognition theoretical model and entropy method were combined to assess water quality in the Harbin reach of the Songhuajiang River in China. A dataset consisting of ten parameters was collected from January to October in 2012. Fuzzy rough set was applied to reduce the ten parameters to four parameters: BOD_5_, NH_3_-N, TP, and F. *coli* (Reduct A). Considering that DO is a usual parameter in water quality assessment, another reduct, including DO, BOD_5_, NH_3_-N, TP, TN, F, and F. *coli* (Reduct B), was obtained. The assessment results of Reduct B show a good consistency with those of Reduct A, and this means that DO is not always necessary to assess water quality. The results with attribute reduction are not exactly the same as those without attribute reduction, which can be attributed to the α value decided by subjective experience. The assessment results gained by the fuzzy rough set obviously reduce computational complexity, and are acceptable and reliable. The model proposed in this paper enhances the water quality assessment system.

## 1. Introduction

As human activities have intensified in recent years, water pollution has become more and more serious and drawn much local and international attention [1,2,3,4]. High attention to water quality protection has a positive effect on water quality assessment, which is an effective way to provide theoretical support for water resource protection. There are lots of methods for water quality assessment, such as matter element analysis [5], multivariate statistical techniques [6,7], artificial neural network [8], Dempster-Shafer evidence theory [9], fuzzy synthetic evaluation [10,11], water quality index [12], and TOPSIS method [13,14], making it difficult to decide which method is the best [14], but it is highly important to choose a method that suits the specific objectives. The attributes recognition theoretical model (ARTM) proposed by Cheng is developed based on fuzzy theory [15]. Fuzzy synthetic evaluation is a common method used in comprehensive multi-attribute assessment. However, environment quality assessment is a problem of ordered partition class, which results in the inappropriate use of the maximum membership principle in fuzzy synthetic evaluation [15], and the maximum membership principle may cause unreasonable assessment results. Considering the characteristics of water quality assessment and the concept of ordered partition class in ARTM, in this study ARTM is selected to assess water quality.

The determination of weights is a vitally significant aspect of water quality assessment, as the weights of parameters can obviously affect assessment results. Therefore, how to choose an appropriate determination method has received enhanced awareness. A large number of weight determination methods are introduced to assess water quality [5,10,16,17]. The entropy method is an objective way to calculate parameter weights. In information theory, entropy can measure the amount of information provided by a system. According to the variation degree of parameter values, information entropy is employed to determine the parameter weight. The entropy weight of the parameter becomes smaller with the increase of the information entropy. A parameter with an information entropy value of 1, which means the parameter provides no effective information to decision makers, can be eliminated [11,18]. In this study, the entropy method is introduced to determine the weights of water quality parameters because of its objectivity and simplicity.

Besides the determination of weights, the selection of parameters is another important issue in water quality assessment. A large amount of parameters are obtained during water quality monitoring, yet, all the parameters are not equally important, and some parameters are even irrelevant to the assessment results. If all the parameters monitored are used to assess water quality, the computation will definitely be complicated. It is usual to choose parameters based on subjective experience to reduce the input space dimensions, but this is not reasonable and is unreliable to some extent. In order to be objective, Principal Component Analysis (PCA) and Factor Analysis (FA) are used to reduce the input space dimensions [19,20]. However, the number of objects should be double or triple that of parameters. The rough set (RS) approach is introduced to reduce the input dimensions with small samples and multiple parameters. RS, originally proposed by Pawlak, is a mathematical tool to handle vagueness and uncertainty information [21]. Attribute reduction is one importation application of RS. RS attribute reduction involves finding out the subsets of the original dataset without changing the objects classification, where the dataset contains discrete attribute values. Nevertheless the pure rough set (PRS) tool is not good at coping with real valued attributes, and the water quality monitoring data are real attribute values. To solve this problem, real valued attributes should be discretized to be symbolic valued attributes. It is generally accepted that to discretize data will cause information loss. Another way to resolve the problem is using a fuzzy rough set (FRS), in which a fuzzy set is combined into a rough set. However, PRS and FRS are not good at handling noisy data. In practice, noise exists in real-world applications and comes from many sources. The occurrence of noisy data should be tolerable by any model constructed. Therefore, the variable precision rough set (VPRS) concept is introduced to cope with uncertain data [22]. VPRS is an extension of RS [21,23], designed to resolve uncertainty problems with an error-tolerance capability [24]. FRS is applied in various areas [25,26,27,28]. However, applications of RS, especially that of FRS, to water quality assessment are scant [14,29]. In this paper, VPRS is applied to perform parameter attribute reduction before water quality assessment, ARTM is used to assess water quality, and the entropy method is used to decide the weights of parameters.

## 2. Materials and Methods

### 2.1. Water Quality Samples

Songhuajiang River, with a total length of 1,657 km and a drainage area of about 556,800 km^2^, is located between 41°42′ to 51°48′ latitude north and 119°52′ to 132°31′ longitude east. The total runoff is 75.9 billion m^3^. Its headstream includes the southern source and the northern source. The southern source, the Second Songhuajiang River, originates from Heaven Lake in Jilin Province, and the northern source, Nenjiang River, originates from the southern slopes of the middle part of Yilehuli Mountain, a branch of China’s Great Hinggan Mountains. After the convergence of the southern source and the northern source at Sanchahe Town in Fuyu City, the river is called Songhuajiang River (Songhuajiang main stream) and runs eastwardly until it finally empties into Heilongjiang River in Tongjiang City. Songhuajiang River has a long icebound season, and two flood seasons, the spring flood season and the summer flood season. Harbin station, the major station after the convergence of Second Songhuajiang River and Nenjiang River, is situated at the midstream of Songhuajiang River. Songhuajiang River is the source of water and the receiving water body of wastewater for Harbin City, the capital city of Heilongjiang Province. 

The data for the Harbin reach of January to October in 2012 were chosen as the research target [30]. Each month, ten parameters were selected: pH, dissolved oxygen (DO), chemical oxygen demand by KMnO_4_ (COD_Mn_), chemical oxygen demand (COD), 5-day biochemical oxygen demand (BOD_5_), ammonia nitrogen (NH_3_-N), total phosphorus (TP), total nitrogen (TN), fluoride (F), and fecal coliforms (F. *coli*). According to the attribution of every parameter, these parameters can be divided into three types: efficiency type, cost type, and interval type. Efficiency type means it is best when the parameter value is the biggest; cost type means it is best when the value is the smallest; interval type means it is best when the value is within a certain interval. Among the selected parameters, DO is efficiency type, pH is interval type, and all the other parameters are cost type.

### 2.2. Fuzzy Rough Set Attribute Reduction

An information system represented by a table should be firstly constructed. In the table, a set of objects are depicted by a set of attributes [21]. An information system is defined as:

*S* = (*U*, *A*, *V*, *f*) (1)

where U = {x_1_, x_2_, …, x_m_} is a non-empty finite set of objects, A = {a_1_, a_2_, …, a_n_} is a non-empty finite set of attributes, 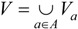 is the value set of attribute *a*, f: U × A → V is an information function, given by the expression (∀(*x*,*a*) ∈ *U* × *A*, *f* (*x*,*a*) ∈ *V_a_*). The FRS attribute reduction steps can be expressed as follows [26,27]:

Step 1. Standardization of the initial data.

Suppose that there are m objects and n parameters to form R as below:

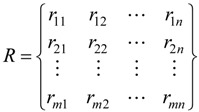
(2)

where R is the initial decision matrix, r_ij_ (*i* = 1, 2, …, m; *j* = 1, 2, …, n) is the observed values.

For efficiency type, the function of standardization is:

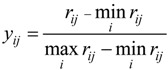
(3)

For cost type, the function of standardization is:

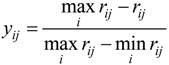
(4)

For interval type, the function of standardization is:

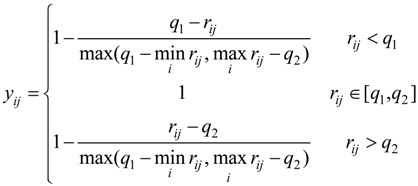
(5)

where [q_1_, q_2_] is the best interval of r_ij_.

After normalization of R, the standard-grade matrix Y can be obtained as:

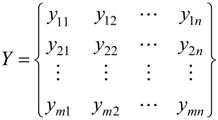
(6)

Step 2. Determination of fuzzy similarity class.

∀*x_s_, x_t_* ∈ *U*, fuzzy similarity relation of x_s_Rx_t_ is defined as:

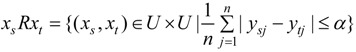
(7)

where α is the distance between x_s_ and x_t_, and 1-α is the similarity degree of x_s_ and x_t_. The value α was set to 0.3 in this study [26]. FR(x_i_), fuzzy similarity class of x_i_, can be got by calculating all the objects that are fuzzy similar to x_i_:

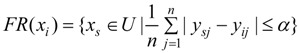
(8)

Step 3. Calculation of lower approximation of variable precision rough set.

PRS attribute reduction relies on lower approximation, which is based on set inclusion. It is sufficient in many applications, but noisy data exist in the real world. To relax the restrictive lower approximation, VPRS is introduced. VPRS can solve classification problems with uncertain data by setting a confident threshold value β. The purpose of VPRS is to classify the objects with a permissible error no greater than a certain pre-defined level. 

Let X be the objects classification of all the parameters, and let FR(a_i_) be the objects classification without the parameter a_i_. X and FR(a_i_) can be obtained by Equation (8). Set confidence threshold value β (0.5 < β ≤ 1) be a real number, the lower approximation of VPRS is defined as:

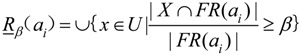
(9)

where |·| denotes cardinality of the set, and the set *R_β_*(*a_i_*) is the set of objects in U that can be classified into X with error classification rate not greater than β. Confidence threshold β was set to be 0.9 in this paper [26].

Step 4. Calculation of β-approximate classification quality.

The β-approximate classification quality is shown as:

*γ_R_*(*a_i_*) = |*R_β_*(*a_i_*)| / |*U*| (10)

To itself, the β-approximate classification quality of the classification by all attributes equals 1. If the classification after eliminating the attribute a_i_ is the same as that before attribute reduction, the β-approximate classification quality should be 1 too. Therefore, based on the β-approximate classification quality, attribute reduction involves ensuring that *γ_R_*(*a_i_*) equals to 1, so the original set is decreased and then the subset of the attributes is obtained [26].

### 2.3. Entropy Method

Entropy method is an objective tool to determine weights of parameters by calculating the difference degree of all parameters. It is calculated as follows [11].

Information entropy should be firstly calculated as:

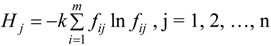
(11)

where H_j_ is the information entropy of the jth parameter, 
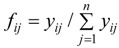
, *k* = 1/ln *m*. When *f_ij_* = 0, assume that *f_ij_* ln *f_ij_* = 0.

Then the entropy weight of the jth parameter is:

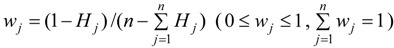
(12)

### 2.4. Attribute Recognition Theoretical Model

The specific steps of ARTM are stated as follows [31,32,33,34].

Step 1. Establishment of attribute space matrix.

There are m objects and n parameters in object space R:

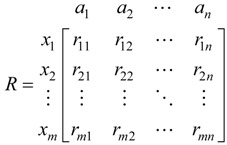
(13)

Suppose F is some attribute space, and (C_1_, C_2_, …, C_K_) is an ordered series of ranks in attribute space F, satisfying C_1_ > C_2_ > … > C_K_. Therefore, the classification standard for each parameter is known, the classification standard matrix can be expressed as A:

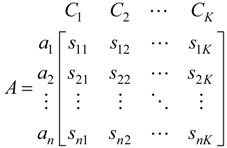
(14)

where *s_j_*_1_ < *s_j_*_2_ < ⋯ < *s_jK_* or *s_j_*_1_ > *s_j_*_2_ > ⋯ > *s_j__K_*.

Step 2. Determination of attribute measure.

The attribute measure *μ_ijk_* = *μ*(*r_ij_* ∈ *C_K_*) of parameter value r_ij_, which takes the attribute levels from the set C_K_, is calculated. Suppose that *s_j_*_1_ < *s_j_*_2_ < ⋯ < *s_jK_*, then:

when *r_ij_* ≤ *s_j_*_1_, assume that *μ_ij_*_1_ = 1, *μ_ij_*_2_ =⋯= *μ_ijK_* = 0;

when *r_ij_* ≤ *s_jK_*, assume that *μ_ijK_* = 1, *μ_ij_*_1_ =⋯= *μ_ijK_*_−1_= 0;

when *s_j_*_l_ ≤ *r_ij_* ≤ *s_j_*_l+1_, assume that

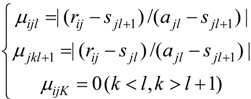
(15)

Considering the weights, the attribute measure of x_i_ is shown as:

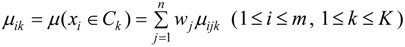
(16)

Step 3. Establishment of attribute recognition theoretical model.

The confidence level *λ* (0.5 ≤ *λ* ≤ 1) is used to determine the rank of x_i_ and described as below:

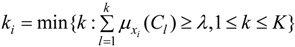
(17)

In the formula, x_i_ is taken to belong to C_k_i__ The confidence level *λ* was set to be 0.75 in this paper [34].

## 3. Results and Discussion

### 3.1. Statistical Analysis

The Environmental Quality Standards for Surface Water of China (EQSSWC) are listed in Table 1. From Table 1, surface water quality in China is classified into five ranks. Ranks I–V are excellent water quality, good water quality, medium water quality, poor water quality, and extremely poor water quality, respectively. Ranks I–III water can be used as the source of drinkable water. Rank III water is used for aquiculture, swimming, and drinking. It is taken as permissible limits in this study (Table 2). The basic statistics of the 10-month dataset on water quality are summarized to give initial information about the Harbin reach of the Songhuajiang River (Table 2). 

**Table 1:** Environmental Quality Standards for Surface Water of China.

Parameters	I	II	III	IV	V
pH	6–9				
DO (mg/L)	≥7.5	≥6	≥5	≥3	≥2
COD_Mn_ (mg/L)	≤2	≤4	≤6	≤10	≤15
COD (mg/L)	≤15	≤15	≤20	≤30	≤40
BOD_5_ (mg/L)	≤3	≤3	≤4	≤6	≤10
NH_3_-N (mg/L)	≤0.15	≤0.5	≤1.0	≤1.5	≤2.0
TP (mg/L)	≤0.02	≤0.1	≤0.2	≤0.3	≤0.4
TN (mg/L)	≤0.2	≤0.5	≤1.0	≤1.5	≤2.0
F (mg/L)	≤1.0	≤1.0	≤1.0	≤1.5	≤1.5
F. *coli* (cfu/L)	≤200	≤2,000	≤10,000	≤20,000	≤40,000

As it can be seen in Table 2, the mean or median values of all studied parameters comply with the requirements set by the permissible limits, with the exception of TN, which is found to be a serious pollutant during the study period. 

pH and the concentration of F are found within the permissible limits. It can also be concluded that F. *coli* has the biggest coefficient of variation (CV), followed by TP, while pH has the smallest. This demonstrates that F. *coli* and TP change a lot from month-to-month, while pH is temporally stable Except for F. *coli*, TP, and pH, the other parameters possess medium CVs, which reveals their concentrations do not change as much as F. *coli* and TP, but more than pH.

**Table 2:** Statistical analysis results for various parameters.

Parameters	Min–Max	Median	Mean	SD	CV	Permissible Limits	MNEPL ^a^
pH (a_1_)	7.16–8.55	7.52	7.61	0.401	0.0527	6–9	0
DO (a_2_)	4.8–13	7.7	8.44	2.6073	0.3089	≥5	1
COD_Mn_ (a_3_)	3.12–6.48	5.04	5.209	0.9733	0.1868	≤6	2
COD (a_4_)	12–23	16.5	16.8	3.49	0.2077	≤20	1
BOD_5_ (a_5_)	1–4.6	2.4	2.69	1.4255	0.5299	≤4	3
NH_3_-N (a_6_)	0.12–1.07	0.44	0.535	0.3868	0.7229	≤1.0	2
TP (a_7_)	0.04–0.69	0.07	0.144	0.1978	1.3738	≤0.2	1
TN (a_8_)	1.1–2.58	1.55	1.607	0.4423	0.2752	≤1.0	10
F (a_9_)	0.24–0.38	0.3	0.298	0.0419	0.1404	≤1.0	0
F. *coli* (a_10_)	20–24,196	1,514	3,793.4	7,227.91	1.9054	≤10,000	1

Note: ^a^ monthly numbers exceeding the permissible limits.

Table 2 reveals that TN is the most main pollution factor. The high concentration of TN often causes algae blooms [35]. TN concentration in a river is the sum of the concentrations of organic nitrogen, nitrate, nitrite, and NH_3_-N. The high concentrations of nitrate, nitrite and NH_3_-N in drinkable water and water source can be poisonous to human and aquatic life. NH_3_-N concentrations beyond the permissible limit lower the oxygen combining ability of aquatic life forms. Fortunately, the NH_3_-N concentration is fairly good and reasonably satisfactory, with only two months showing values slightly higher than the permissible limit. Because Harbin City is the capital city of Heilongjiang Province, and the Songhuajiang River is the receiving water body of wastewater from Harbin City, the high concentration of TN is mainly attributed to domestic sewage and industrial effluents.

**Figure 1:**
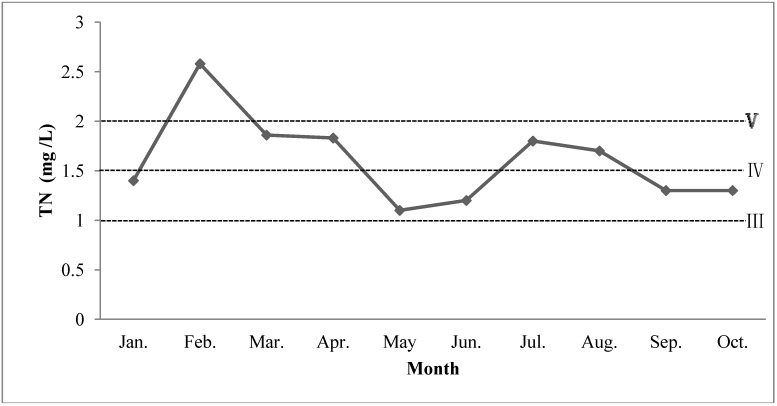
Plot of TN temporal distribution.

TN concentration in the study period is illustrated in Figure 1. Ranks III-V in EQSSWC (Table 1) are marked as dotted lines. TN concentrations in ten months are beyond the permissible limit (1.0 mg/L). The lowest TN concentration is 1.1 mg/L in May, while the highest TN concentration is 2.58 mg/L in February. TN reduction should be a big concern to prevent further pollution in the study area.

### 3.2. Parameters Attribute Reduction

FRS attribute reduction is carried out by MATLAB 8.0. The FRS attribute reduction process is shown in Table 3.

**Table 3:** Process of FRS attribute reduction.

Subset of Reserved Attributes	Subset of Deleted Attributes	β-Approximate Classification Quality	Delete ^a^
{a_2_,a_3_,a_4_,a_5_,a_6_,a_7_,a_8_,a_9_,a_10_}	{a_1_}	1	Y
{a_3_,a_4_,a_5_,a_6_,a_7_,a_8_,a_9_,a_10_}	{a_1_,a_2_}	1	Y
{a_4_,a_5_,a_6_,a_7_,a_8_,a_9_,a_10_}	{a_1_,a_2_,a_3_}	1	Y
{a_5_,a_6_,a_7_,a_8_,a_9_,a_10_}	{a_1_,a_2_,a_3_,a_4_}	1	Y
{a_6_,a_7_,a_8_,a_9_,a_10_}	{a_1_,a_2_,a_3_,a_4_,a_5_}	0.7	N
{a_5_,a_7_,a_8_,a_9_,a_10_}	{a_1_,a_2_,a_3_,a_4_,a_6_}	0.2	N
{a_5_,a_6_,a_8_,a_9_,a_10_}	{a_1_,a_2_,a_3_,a_4_,a_7_}	0.9	N
{a_5_,a_6_,a_7_,a_9_,a_10_}	{a_1_,a_2_,a_3_,a_4_,a_8_}	1	Y
{a_5_,a_6_,a_7_,a_10_}	{a_1_,a_2_,a_3_,a_4_,a_8_,a_9_}	1	Y
{a_5_,a_6_,a_7_}	{a_1_,a_2_,a_3_,a_4_,a_8_,a_9_,a_10_}	0.6	N

Notes: ^a^ whether to delete the new attribute in the subset of deleted attributes, Y (Yes), N (No).

From Table 3, it is shown that {a_5_, a_6_, a_7_, a_10_} is one of the minimum subsets, which will not change the objects classification of the original attributes. The subset of {a_2_, a_3_, a_4_, a_5_, a_6_, a_7_, a_8_, a_9_, a_10_} is utilized to show the process of attribute reduction. The attribute a_1_ is not included in the subset. The fuzzy similarity class of all attributes is shown as X: 

X = {{x_1_,x_2_,x_3_},{x_1_,x_3_,x_5_,x_10_},{x_2_,x_3_,x_4_},{x_3_,x_4_,x_10_},{x_4_,x_8_,x_10_},{x_5_,x_6_,x_10_},{x_6_,x_8_,x_10_},{x_7_,x_8_,x_10_},{x_9_}}

Considering the subset {a_2_,a_3_,a_4_,a_5_,a_6_,a_7_,a_8_,a_9_,a_10_}, fuzzy similarity class can be obtained as FR(a_1_):

FR(a_1_) = {{x_1_,x_2_,x_3_},{x_1_,x_3_,x_5_},{x_1_,x_5_,x_10_},{x_3_,x_4_},{x_4_,x_10_},{x_5_,x_6_,x_10_},{x_7_,x_8_,x_10_},{x_9_}}

The β-approximate classification quality of the subset equals to 1, which means a_1_ can be deleted without affecting objects classifications.

By the same method, the subsets of {a_3_, a_4_, a_5_, a_6_, a_7_, a_8_, a_9_, a_10_}, {a_4_, a_5_, a_6_, a_7_, a_8_, a_9_, a_10_}, {a_5_, a_6_, a_7_, a_8_, a_9_, a_10_}, and {a_6_, a_7_, a_8_, a_9_, a_10_} are calculated. It is found that the β-approximate classification quality of the subset {a_6_, a_7_, a_8_, a_9_, a_10_} is not equal to 1. This indicates that the attribute a_5_ cannot be deleted.

Finally, one reduct {a_5_, a_6_, a_7_, a_10_} (Reduct A) can be obtained. There is always more than one reduct in RS attribute reduction. Because DO is taken as an important parameter to assess water quality, another reduct {a_2_, a_5_, a_6_, a_7_, a_8_, a_9_, a_10_} (Reduct B) is gained to compare with Reduct A.

Because the value α in fuzzy similarity relation is set by subjective experience, different α values are assigned to obtain other reducts to discuss the effect of the value α. The reducts {a_4_, a_6_, a_7_, a_8_} (Reduct C), {a_3_, a_6_, a_7_, a_8_, a_9_, a_10_} (Reduct D), {a_4_, a_5_, a_6_, a_7_, a_9_} (Reduct E), and {a_4_, a_5_, a_6_, a_7_} (Reduct F) are obtained when α is set to be 0.29, 0.28, 0.27, and 0.26/0.25, respectively. The same reduct (Reduct F) can be obtained when α is 0.26 and 0.25. 

### 3.3. Weights of Parameters

Using the calculation method in Equation (11), the information entropy of the four parameters can be obtained. Then according to Equation (12), each parameter gets a weight. The information entropy and weight of each parameter are revealed in Table 4. 

**Table 4:** Weights of parameters calculated by entropy method.

Parameters	Information Entropy	Weight
BOD_5_	0.8617	0.3701
NH_3_-N	0.8579	0.3802
TP	0.9528	0.1263
F. *coli*	0.9539	0.1234

### 3.4. Water Quality Assessment

After calculating the entropy weights of the four parameters after FRS attribute reduction, ARTM is applied to assess water quality in the Harbin reach of the Songhuajiang River and the results of Reduct A are shown as Reduct A in Table 5. Reduct A includes the parameters of BOD_5_, NH_3_-N, TP and F. *coli*. In China, DO is a usual parameter used to assess water quality. Reduct B, including the parameters of DO, BOD_5_, NH_3_-N, TP, TN, F, and F. *coli*, is obtianed to compare with Reduct A. The assessment results of Reduct B are presented as Reduct B. In addition, the results of Reducts C–F are described as Reduct C, Reduct D, Reduct E, and Reduct F, respectively.

**Table 5:** Assessment results of the Harbin reach of the Songhuajiang River.

Methods	Reducts	Jan.	Feb.	Mar.	Apr.	May	Jun.	Jul.	Aug.	Sep.	Oct.
With attribute reduction	Reduct A	Ⅲ	Ⅲ	Ⅲ	Ⅲ	Ⅲ	Ⅱ	Ⅱ	Ⅱ	Ⅳ	Ⅱ
	Reduct B	Ⅲ	Ⅲ	Ⅲ	Ⅲ	Ⅲ	Ⅱ	Ⅱ	Ⅱ	Ⅳ	Ⅱ
	Reduct C	Ⅲ	Ⅲ	Ⅲ	Ⅲ	Ⅱ	Ⅱ	Ⅲ	Ⅲ	Ⅳ	Ⅱ
	Reduct D	Ⅲ	Ⅲ	Ⅲ	Ⅲ	Ⅱ	Ⅲ	Ⅲ	Ⅲ	Ⅲ	Ⅲ
	Reduct E	Ⅲ	Ⅲ	Ⅲ	Ⅲ	Ⅲ	Ⅱ	Ⅱ	Ⅱ	Ⅲ	Ⅱ
	Reduct F	Ⅲ	Ⅱ	Ⅲ	Ⅲ	Ⅲ	Ⅱ	Ⅱ	Ⅱ	Ⅳ	Ⅱ
Without attribute reduction		Ⅲ	Ⅲ	Ⅲ	Ⅲ	Ⅲ	Ⅱ	Ⅲ	Ⅲ	Ⅲ	Ⅱ

Table 5 reveals that the water quality in the Harbin reach of the Songhuajiang River is generally acceptable during the study period. The assessment results without attribute reduction show that June and October are good quality water (Rank II), and the other months are medium quality water (Rank III). While, the assessment results (Reducts A–F) show that all objects are good quality water (Rank II) or medium quality water (Rank III) except September (Rank IV for Reducts A to C and F).

The results with attribute reduction (Reducts A–F) are not exactly the same as those without attribute reduction. There are three objects in Reduct A, Reduct B, and Reduct D, two objects in Reduct C and Reduct E, and four objects in Reduct F, whose ranks are different from those without attribute reduction. The differences can be attributed to the selection of the value *α*. The value *α* chosen by subjective experience is a measure for the distance of two objects. The value 1-*α* is the similarity degree of the two objects. In theory, the similarity degree of the two objects becomes bigger with the decrease of the value *α*. It is difficult to find fuzzy similarity classes with smaller *α* value, while it becomes useless to find fuzzy similarity classes with bigger *α* value. Hence, the selection of the value α is very important, and the appropriate value α can narrow the gap between the results before attribute reduction and the results after attribute reduction. The value α in fuzzy similarity relation does have effect on the assessment results. Although the results with attribute reduction are somewhat different from those without attribute reduction, the differences are still acceptable. This means that FRS is a good tool to perform attribute reduction and the results are reasonable and reliable.

The results of Reduct A and Reduct B are exactly the same. Reduct A includes the parameters of BOD_5_, NH_3_-N, TP, and F. *coli*, while Reduct B is comprised of the parameters of DO, BOD_5_, NH_3_-N, TP, TN, F, and F. *coli*. The results by Reduct A and Reduct B in this paper seem to indicate that DO is not always necessary to assess water quality. In fact, DO concentration is sufficient in the Songhuajiang River owing to its fluidity.

## 4. Conclusions

In this study, a fuzzy set was combined with a rough set to perform attribute reduction of water quality parameters, because of the limitations of the pure rough set. An entropy method was used to calculate the parameter weights. The attribute recognition theoretical model was successfully applied to evaluate water quality rankings for the period from January to October in 2012 for the Harbin reach of the Songhuajiang River in China. The results indicate that water quality in study area is acceptable. Nevertheless, special attention should be paid to prevent further water pollution. For example, TN is the major pollutant factor in the study area, and TN concentrations in ten months exceeded the permissible limit (Rank III), with one month beyond Rank V. A fuzzy rough set was employed to handle the water quality data to perform attribute reduction. After attribute reduction, the assessment results are almost the same as those from before attribute reduction. This shows that that fuzzy rough set theory is a reasonable and reliable way to perform attribute reduction. Especially for datasets with a large number of parameters and small objects, the fuzzy rough set can obviously reduce input space dimensions and computation complexity. However, there are still some objects with attribute reduction showing different results from those without attribute reduction, which perhaps can be attributed to the value α decided by subjective experience. The assessment results of five reducts (Reduct A, Reduct C, Reduct D, Reduct E, and Reduct F) are somewhat different from those without attribute reduction. The differences can be accepted. Determining how to select the value α to get reducts is very important in this paper, and it will be discussed in our future study. Although the assessment results with attribute reduction are not perfect now and still need improvement, the fuzzy rough set can still be regarded as a useful tool to perform attribute reduction to reduce input space dimensions.
